# Photobiomodulation Drives MiR-136-5p Expression to Promote Injury Repair after Myocardial Infarction

**DOI:** 10.7150/ijbs.71440

**Published:** 2022-04-18

**Authors:** Xinlu Gao, Hanjing Li, Wenwen Zhang, Xiuxiu Wang, Hongyue Sun, Yang Cao, Yiming Zhao, Haoyu Ji, Fan Yang, Wenya Ma, Yu Liu, Baofeng Yang, Benzhi Cai

**Affiliations:** 1 Department of Laboratory Medicine at the Fourth Affiliated Hospital, and Department of Pharmacy at the Second Affiliated Hospital, Harbin Medical University, Harbin, China; 2 Department of Pharmacology (State-Province Key Laboratories of Biomedicine-Pharmaceutics of China, Key Laboratory of Cardiovascular Research, Ministry of Education) at College of Pharmacy, Harbin Medical University, Harbin, China; 3 Northern Translational Medicine Research and Cooperation Center, Heilongjiang Academy of Medical Sciences, Harbin Medical University, Harbin, ChinaHarbin, China; 4 Institute of Clinical Pharmacy, the Heilongjiang Key Laboratory of Drug Research, Harbin Medical University, Harbin, China

**Keywords:** Photobiomodulation therapy, Light-emitting diodes, Cardiomyocyte proliferation, Myocardial regeneration, Myocardial infarction.

## Abstract

Photobiomodulation (PBM) has emerged as an alternative therapy involved in modulating a variety of biological effects. In this study, we verified whether PBM can affect cardiac physiological activity in mice through noninvasive irradiation using light-emitting diodes at a wavelength of 630 nm (LED-Red). We found that the PBM involved in regulating the repair of injured myocardium is wavelength-limited. LED-Red caused cardiomyocytes (CMs) that had exited the cell cycle to divide and proliferate again, and the cell proliferation ratio increased significantly with the accumulation of intracellular photopower. In addition, LED-Red promoted myocardial revascularization and myocardial regeneration, reduced the area of fibrosis in mice with myocardial infarction (MI), and thus improved cardiac contractile function. In regard to the mechanism, miRNA sequencing analysis showed that low-power LED-Red irradiation could induce differential changes in miRNAs in CMs. Among them, miR-136-5p was identified as a cardiac photo-sensitive miRNA and was obviously inhibited after stimulation, which produced a proliferation-promoting effect on CMs. Subsequent luciferase reporter assays confirmed the involvement of Ino80 as a binding target of miR-136-5p in the regulatory process of CM proliferation. Similarly, LED-Red irradiation elevated intracellular Ino80 expression. After knockdown of Ino80, the proliferation-promoting effect of LED-Red on CMs was inhibited. Collectively, this study demonstrates that LED-Red can promote CM proliferation by inhibiting cardiac photo-sensitive miRNA- miR-136-5p expression through targeting Ino80. The findings provided a new potential strategy for the treatment of ischemic cardiomyopathy (ICD).

## Introduction

Ischemic cardiomyopathy, mainly MI, remains one of the diseases causing the highest mortality rates worldwide 1. The mammalian heart gradually withdraws from the CM proliferation cycle after birth, maintaining the low self-renewal state and hardly undergoing cell proliferation 2. Therefore, the adult myocardial damage caused by MI is almost irreversible due to the loss of CMs. Recently, an increasing number of studies have shown that mammalian heart tissue can activate quiescent CM into a proliferative state after receiving exogenous stimulation 3-5. Among the numerous regulators identified for the re-entry of the CM proliferation cycle, microRNAs (miRNAs) are also thought to play an important role in this process 6-8. Despite scientific progress in uncovering the molecular mechanisms involved in the pathological process of MI, effective therapeutic approaches are still limited. Therefore, further exploration of a new therapeutic approaches is necessary.

Photobiomodulation therapy (PBMT) refers to low-power (1-500 mW) non-thermal transfer photons with a spectrum in the visible or near-infrared (400-1000 nm) range that trigger beneficial biological responses in cells and tissues 9. Red and near-infrared light are the main light sources for PBMT. Several studies have shown that red light has the ability to promote the proliferation of both neural stem cells and skeletal muscle satellite cells 10, 11. However, the information about the modulation of the heart by red light is still limited 12, 13. Although recent studies showed that LEDs can affect several biological processes, the effect of PBM on CM proliferation and myocardial regeneration is still unknown. Therefore, the aim of this study was to investigate whether 630 nm LED can modulate CM proliferation and reduce myocardial collagen deposition so as to improve left ventricular systolic function after MI.

## Results

### Effective LED light wavelength and illumination parameters for cardiomyocytes

**Figure 1:**
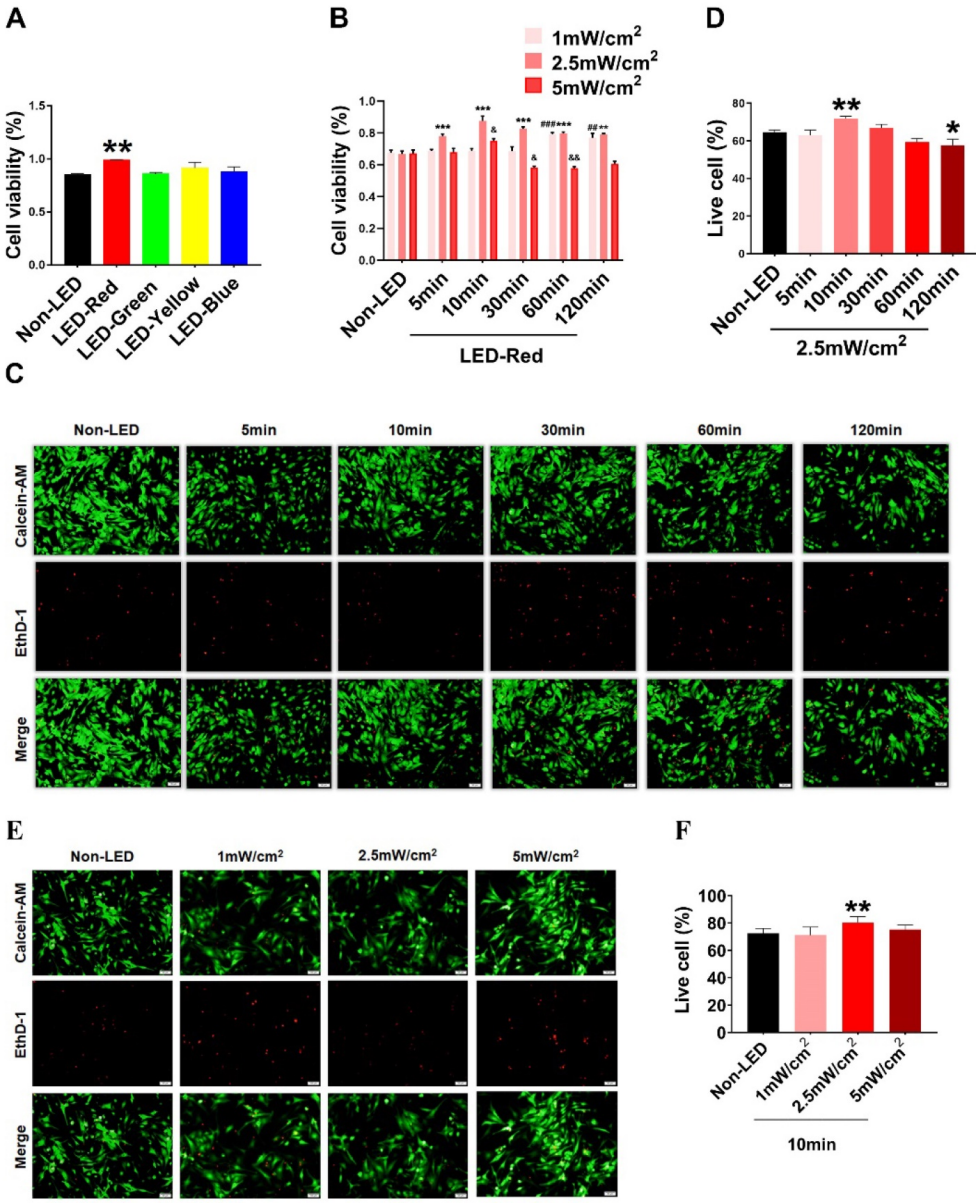
** Optimal effective LED light wavelength and illumination parameters.** CCK-8 assay of CM viability under (A) different wavelengths of light stimulation. (B) LED-Red irradiation at different times and power densities. n = 3. ^&^*P* < 0.05, ^&&^*P* < 0.01, **P < 0.01, ***P < 0.001, ^##^P < 0.01, ^###^*P* < 0.001vs. Non-LED. (C), (E) Live-dead cell staining was used to detect CM phototoxicity after LED-Red irradiation under different parameters. (D), (F) Live cell ratio statistics. *P < 0.05, **P < 0.01 vs. Non-LED. n = 3. Scale bar = 50 μm. The data represent the mean ± SEM.

Based on previous studies, PBM regulates a variety of biological processes in a wavelength-dependent manner, and the biological effects produced by light power density and light duration show a bell-shaped distribution 14. Therefore, we explore whether different wavelengths of light stimulation will have different effects on CMs. We first examined the viability of CMs under multiple wavelengths of LEDs, including red (peaked at 630 nm), blue (peaked at 470 nm), green (peaked at 560 nm), and yellow (peaked at 595 nm). The results showed that LED-Red upregulated CM viability compared with non-LED (Figure 1A). Next, to investigate the specific role of LED-Red in CMs, we further explored the effective light conditions for LED-Red to regulate physiological activity of CMs. CMs received LED-Red stimulation at different power densities for 5, 10, 30, 60 and 120 min, respectively. We found that cell viability was maintained at a high level at an LED-Red power density of 2.5 mW/cm^2^. Moreover, the cell viability reached the peak when the irradiation time was 10 min (Figure 1B). Also, we investigated whether light stimulation for 10 min at the three power densities would produce phototoxicity to CMs. The results showed that 2.5mW/cm^2^ condition resulted in an increase of the live cell ratio compared with the Non-LED group (Figure 1E-F). In addition, we investigated the photodamage effect of prolonged exposure with a power density of 2.5 mW/cm^2^. The results showed that extending the stimulation time to 120 min resulted in a decrease in the percentage of live cells, while irradiation for 10 min increased this percentage. Thus, 2.5 mW/cm^2^ for 10 min which can improve CM cell viability and does not cause phototoxicity, is the suitable light irradiation condition for CMs (Figure 1C-D).

### LED-Red irradiation promotes cardiomyocyte proliferation

**Figure 2:**
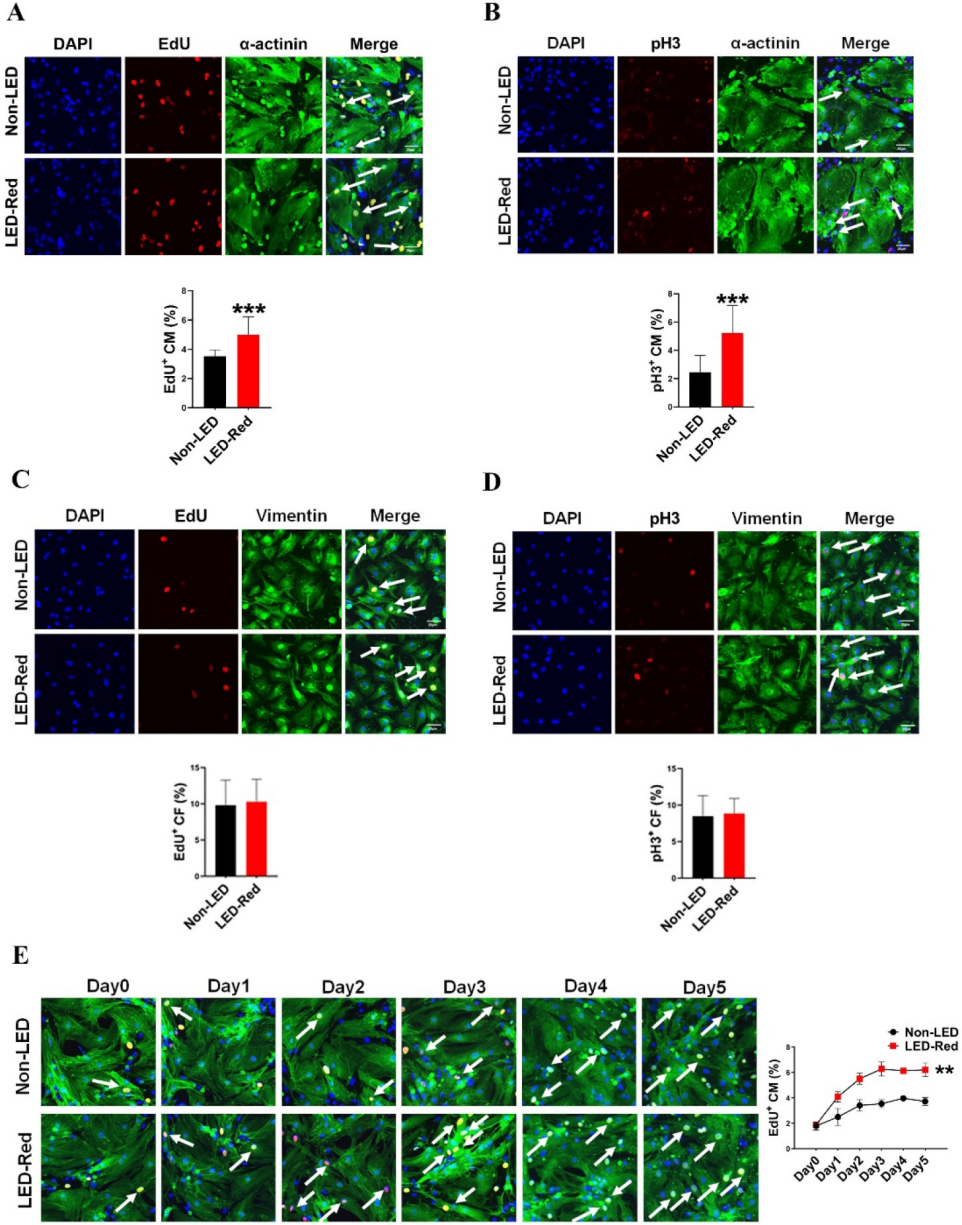
**LED-Red irradiation promotes cardiomyocyte proliferation.** (A), (C) and (E) EdU (B), (D) pH3 detection of CM (or CF) proliferation after LED-Red irradiation. n = 3. Scale bar = 20 μm. The data represent the mean ± SEM. ***P < 0.001 vs. Non-LED.

Red light in the visible range has shown pro-proliferative functions on different types of cells 10, 11, 15. However, the regulatory effects of LED-Red on CM proliferation are still unknown. Therefore, we then verified the relationship between LED-Red and CM proliferation. Immunofluorescence staining for the proliferation marker EdU showed that LED-Red can promote DNA replication of quiescent CMs and re-enter the proliferation cycle (Figure 2A). In addition, another proliferation marker, pH3, similarly demonstrated that CMs exhibited significant mitosis under LED-Red irradiation (Figure 2B). Then, to confirm the cumulative effect of LED-Red on CM proliferation, the cells were exposed to 2.5 mW/cm^2^ LED-Red for 10 min per day for 5 consecutive days, and EdU incorporation assay was used to detected the proliferation rate of CMs. Compared to the time-matched controls, the proliferation rate of CMs showed a positive correlation with the irradiation time. The peak was reached at 3 days of continuous LED exposure (Figure 2E). Collectively, these data suggest that LED-Red can promotes CM proliferation and produce a cumulative pro-proliferative effect on CMs. The cell types in the heart include CMs, endothelial cells (ECs) and fibroblasts (CFs). To verify whether LED-Red has the same regulatory effect on non-cardiomyocytes in the heart, we also examined the proliferation rate of CFs after LED-Red irradiation. The results showed that LED-Red stimulation did not have a pro-proliferative effect on CFs (Figure 2C, D).

### LED-Red enhances heart function in adult mice after MI

**Figure 3:**
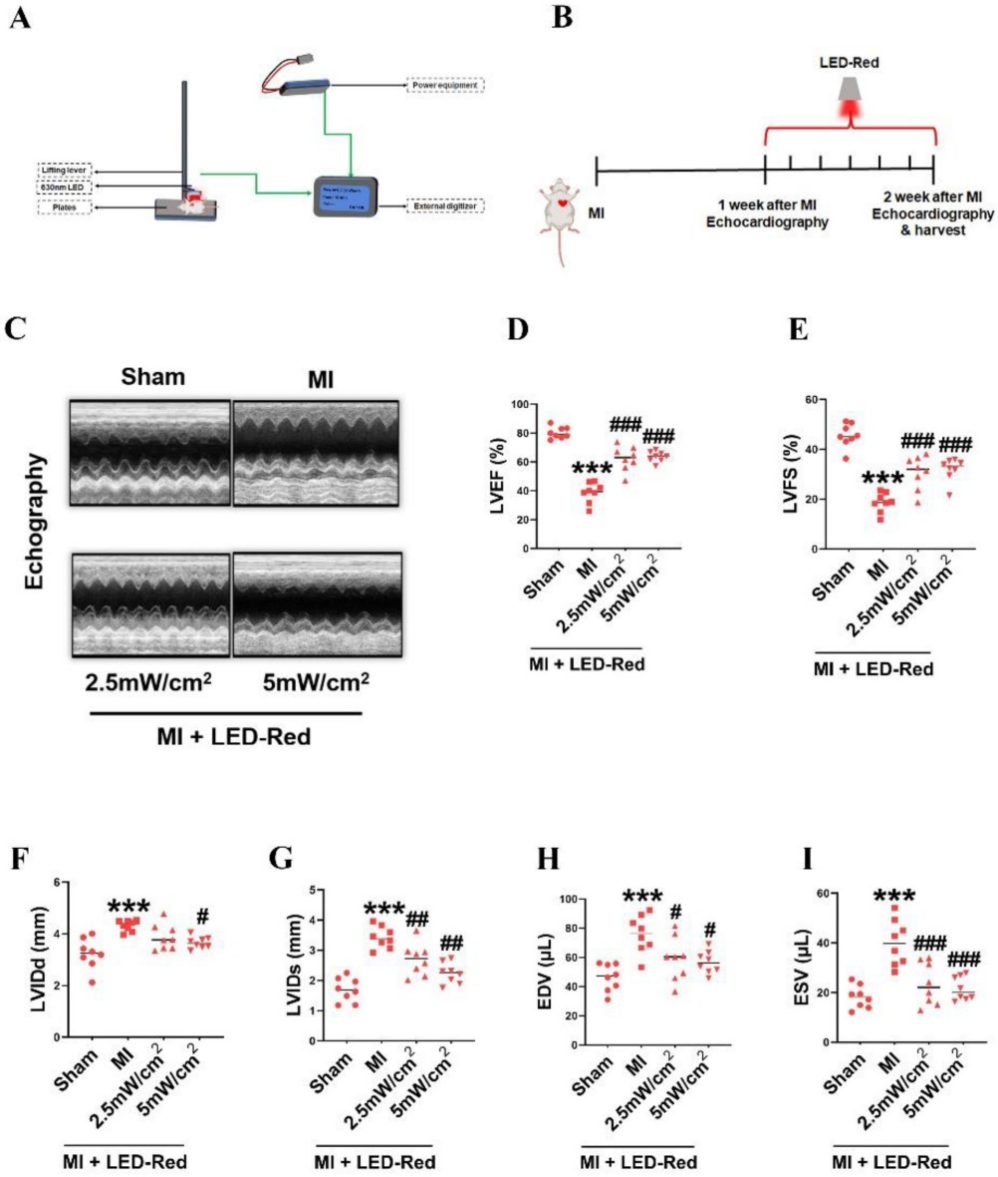
** LED-Red enhances heart function in adult mice after MI.** (A) Animal experiment irradiation pattern diagram. (B) Flow chart of irradiation for animal experiments. (C) Echocardiography (D) LVEF (E) LVFS (F) LVIDd (G) LVIDs (H) EDV (I) ESV. n = 8, The data represent the mean ± SEM. ***P < 0.001 vs. Sham, ^#^*P* < 0.05, ^##^*P* < 0.01, ^###^*P* < 0.001 vs. MI.

Then, we investigated whether LED-Red could repair infarcted myocardial tissue and improve cardiac function by regulating myocardial regeneration. We used a LED monochromatic photosystem to irradiate the mice heart (Figure 3A, S2). As shown in Figure 3A, the light source was placed immediately on the left side of the mice's chest, and the hairs on the left side of the chest were removed. The mice were exposed to LED-Red for 7 days (10 min/day) after left ventricular ligation (Figure 3B). Echocardiography showed that LVEF and LVFS were significantly improved and LVIDd and LVIDs were decreased in the LED-Red group compared with the MI group (Figure 3C-G). Furthermore, LVEDV and LVESV were significantly smaller in the LED-Red group than in the MI mice (Figure 3H, I).

### LED-Red improves myocardial remodeling in adult mice after MI

**Figure 4:**
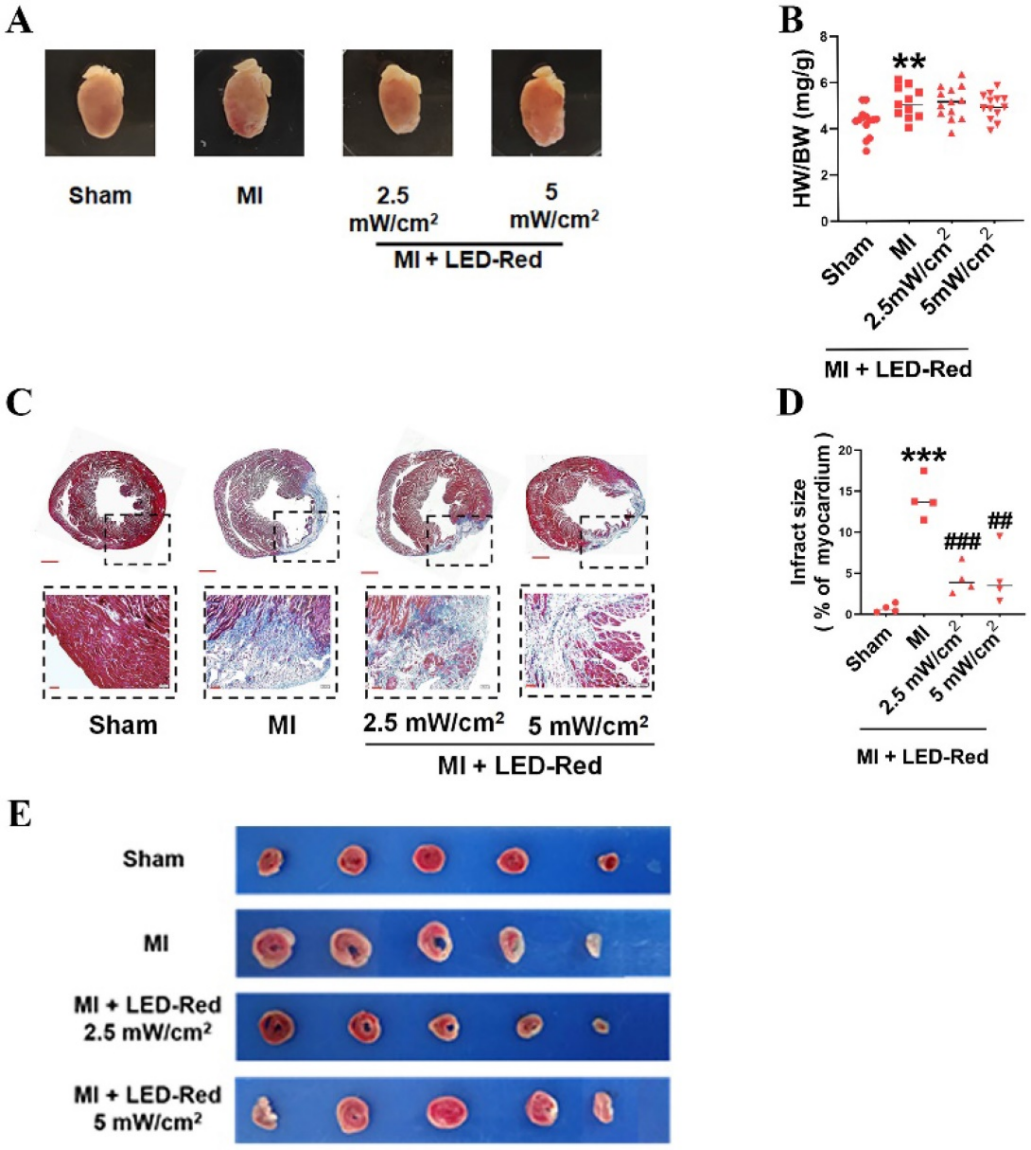
** LED-Red represses myocardial remodeling in adult mice after MI.** (A) Heart morphology. (B) Heart to weight ratio (HW/BW) n = 13. (C), (E) Masson and TTC staining to detect the extent of MI. (D) Statistical graph of MI area. n = 4. Scale bar = 50 μm.The data represent the mean ± SEM. **P < 0.01, ***P < 0.001 vs. Sham, ^##^*P* < 0.01, ^###^*P* < 0.001 vs. MI.

We further observed the influence of LED-Red on myocardial remodeling in adult mice after MI. Morphological and structural analyses showed no significant effect of LED-Red stimulation on heart morphology, volume size, and the heart weight to body weight ratio after MI (Figure 4A, B). TTC and Masson trichrome staining revealed that the degree of myocardial fibrosis was significantly decreased by LED-Red exposure compared to the Non-LED MI group, and the infarcted area was reduced in the LED-Red groups (Figure 4C-E). In summary, LED-Red has a modulation of myocardial contractile function and contractile volume in infarcted mice. Also, it attenuated myocardial collagen deposition and infarct size, but had no significant effects on overall heart size and weight.

### LED-Red promotes heart regeneration in mice after MI

**Figure 5:**
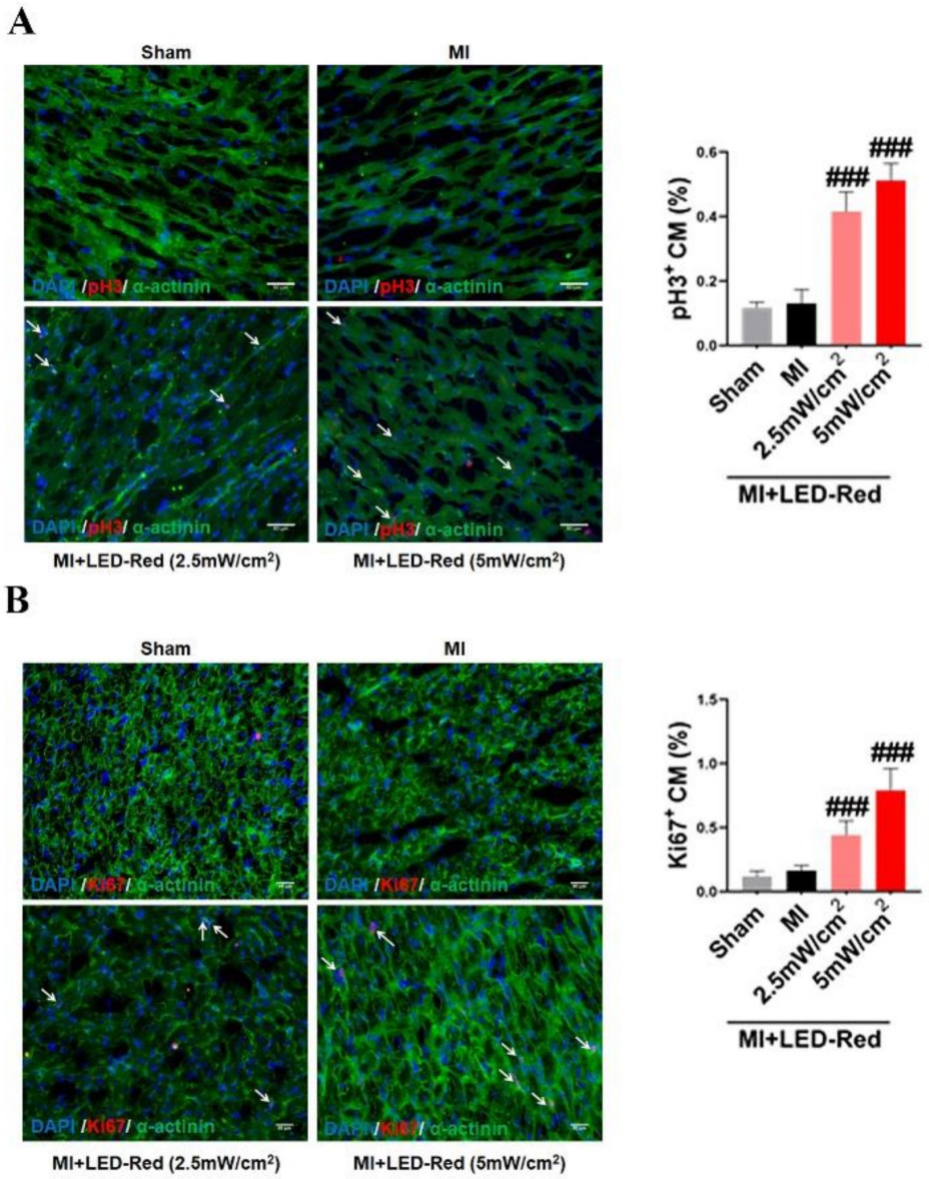
**LED-Red promotes heart regeneration in mice after MI.** Frozen sections of myocardial tissue (A) pH3, (B) ki67 staining for analysis of myocardial regeneration. n = 3. Scale bar = 50 μm. The data represent the mean ± SEM. ^###^*P* < 0.001 vs. MI.

**Figure 6:**
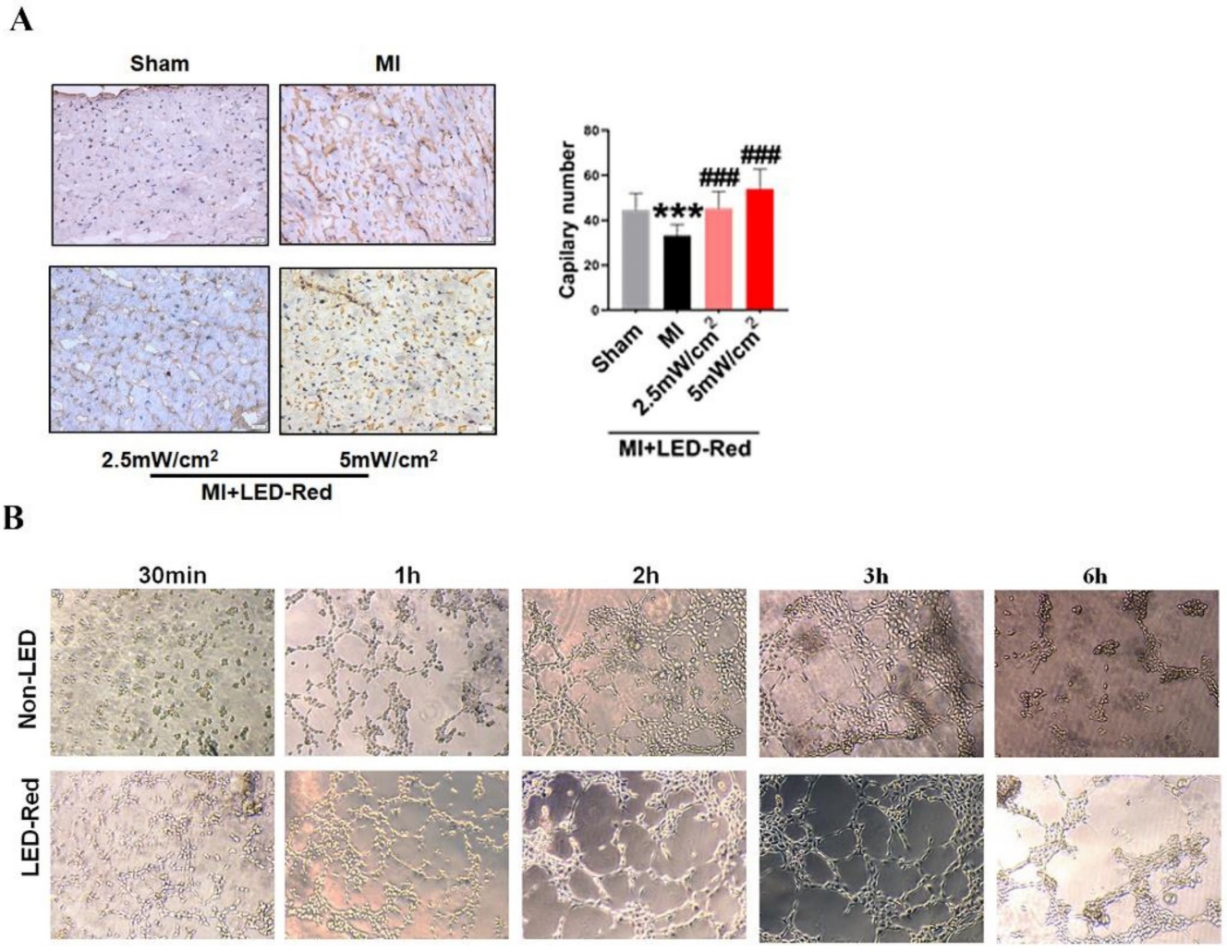
**LED-Red promotes myocardial angiogenesis.** (A) vWF staining to detect myocardial tissue revascularization (B) HUVESCs to verify blood vessels after LED-Red irradiation. n = 3. Scale bar = 50 μm. The data represent the mean ± SEM. ***P < 0.001 vs. Sham, ^###^*P* < 0.001 vs. MI.

Next, we performed immunofluorescence staining for proliferation markers, including pH3 and ki67, on myocardial tissue from LED-Red+MI mice. Interestingly, the proportion of EdU and pH3 positive CMs at the infarct border was significantly increased after LED-Red, compared with mice in the unirradiated MI group (Figure 5A, B). The above results suggest that LED-Red irradiation can effectively promote myocardial regeneration after MI and participate in cardiac function improvement in this way. Several studies have reported that ECs play an essential role in the regulation of cardiac growth and homeostasis via angiogenesis 16. Therefore, we observed and measured the effect of LED-Red on the number of capillaries in the border area of MI. The results showed an increase in capillary density in the border zone after stimulation with LED-Red (Figure 6A). In addition, we also studied the effect of LED-Red on human umbilical vein endothelial cells (HUVESc) tube formation *in vitro* experiments. As shown in Figure 6B, LED-Red accelerated the form of the tube at the beginning of the experiment at the 30 min. It indicates that LED-Red accelerates the repair of post-infarct damage by increasing the formation of blood vessels. Together, the above findings demonstrate that LED-Red promotes myocardial regeneration by regulating cardiomyocyte proliferation and angiogenesis at the infarct border.

### MiR-136-5p as a LED-Red sensitive miRNA promotes the proliferation of cardiomyocyte

**Figure 7:**
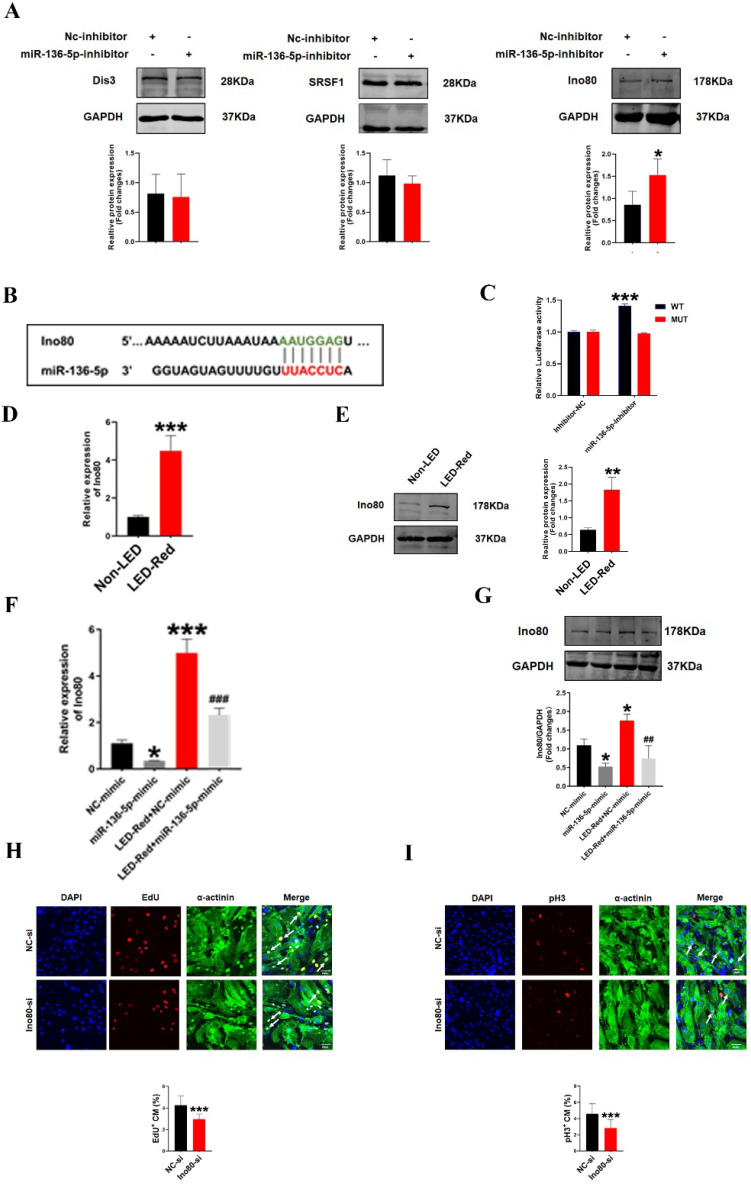
**LED-Red regulates miR-136-5p to promote the proliferation of cardiomyocytes.** miRNA sequencing analysis (A) Heat map (B) Volcano map to detect differentially expressed miRNAs after irradiation by LED-Red. (C) qRT-PCR to verify miRNA expression. **P < 0.01, ***P < 0.001 vs. Non-LED. After knockdown or overexpression of miR-21a-3P and miR-136-5p, respectively, the proliferation of CMs was detected by (D), (F) EdU, (E), (G) pH3 staining. CM proliferation was detected by (H) EdU, (I) pH3 after miR-136-5p-mimic combined with LED-Red treatment. n = 3. Scale bar = 20 μm. *P < 0.05, **P < 0.01, ***P < 0.001 vs. NC-mimic (or inhibitor), ^###^*P* < 0.001 vs. LED-Red+NC-mimic. The data represent the mean ± SEM.

In recent years, many studies have shown that miRNAs can be used to induce CM proliferation and restore cardiac function after MI 17, 18. We thus examined the changes of miRNAs in CMs after irradiation using miRNA sequencing analysis. Notably, 13 miRNAs were upregulated and 4 miRNAs were significantly downregulated by LED-Red irradiation (Figure 7A, B), suggesting these miRNAs are possibly photo-sensitive. Next, we used qRT-PCR to validate miRNAs that underwent differential changes in RNA sequencing analysis. Results showed that the changes of miR-21a-3p, miR-136-5p, and miR-6933-5p were consistent with the miRNA sequencing results (Figure 7C). But, we did not explore the role of miR-6933-5p in the follow-up study because of its low conservation.

Subsequently, we verified the regulatory effects of miR-21a-3p and miR-136-5p on CMs proliferation. CMs were transfected with miR-21a-3p and miR-136-5p inhibitor, respectively. EdU staining demonstrated a significant increase in the proliferation ratio of CMs after inhibition of miR-136-5p expression, but inhibition of miR-21-3p did not significantly affect it (Figure 7D). pH3 staining showed that both miR-21a-3p inhibitor and miR-136-5p inhibitor promoted mitosis in CMs (Figure 7E). In contrast, both miRNAs overexpression showed inhibition of CM proliferation (Figure 7F-G). Because the effects of miR-136-5p on CM proliferation are stronger, in the later studies we mainly explored the role of miR-136-5p in the regulation of CMs proliferation by LED-Red. To investigate the role of miR-136-5p in LED-Red stimulated CMs, the proliferative ability of CMs was detected by EdU and pH3 in CMs. The results showed that overexpression of miR-136-5p impaired the proliferation ability of LED-Red-induced CMs (Figure 7H, I). Collectively, the above results suggest that cardiac photo-sensitive miR-136-5p plays an important role in promoting CMs proliferation. Meanwhile, miR-136-5p could serve as a downstream target of LED-Red to regulate the proliferation cycle of CMs.

### Ino80 is a target of miR-136-5p and regulates cardiomyocyte proliferation following LED-Red stimulation

**Figure 8:**
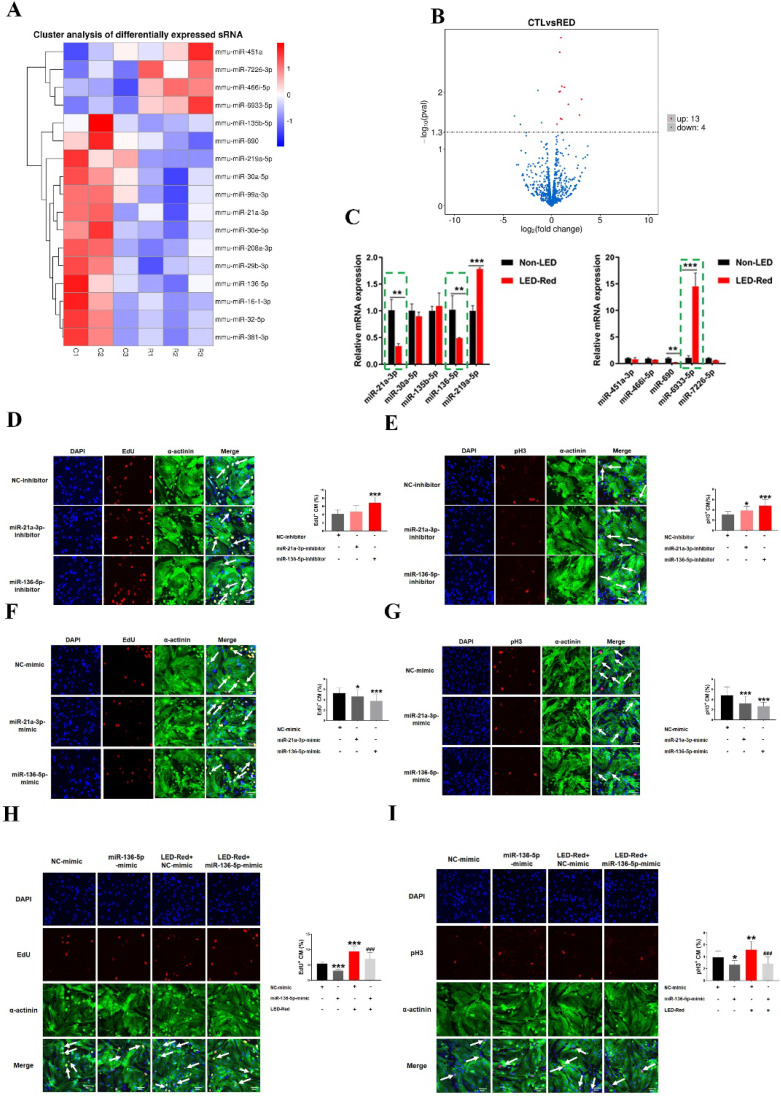
** Ino80 acts as a target of miR-136-5p and regulates cardiomyocyte proliferation following LED-Red stimulation.** (A)Western Blot analysis of Ino80, Dis3 and Srsf1 in CMs transfected with miR-136-5p inhibitor, n = 3, *P < 0.05vs. NC-inhibitor, (B) The predicted binding site of miR-136-5p for Ino80. (C) The luciferase assay was used to analyze the binding between miR-136-5p and Ino80. (D) qRT-PCR and (E) Western Blot were used to detect the expression level of Ino80 in CMs after LED-Red exposure, n = 3, **P < 0.01, ***P < 0.001 vs. Non-LED (F) qRT-PCR and (G) Western Blot to determine the effect of miR-136-5p-mimic combined with LED-Red on Ino80 expression in CMs. *P < 0.05, ***P < 0.001 vs. NC-mimic, ^##^*P* < 0.01, ^###^*P* < 0.001 vs. LED-Red+NC-mimic. The effect of knockdown of Ino80 on CM proliferation was detected using (H) EdU, (I) pH3. Arrows indicate proliferative signals, n = 3, Scale bar = 20 μm. ***P < 0.001vs. NC-si. The data represent the mean ± SEM.

To further investigate the involvement of miR-136-5p in the regulatory role of LED-Red on the proliferation of CMs, we explored the possible targets of miR-136-5p. Target genes of miR-136-5p were predicted by TargetScan Mouse algorithm. We identified Dis3, Srsf1 and Ino80 as known proliferation regulators have binding sites with miR-136-5p at the 3'UTR (Figure 8B, S1). We first detected the protein expression of the three target genes after inhibition of miR-136-5p by Western Blot. The results showed that Ino80 protein levels were remarkably increased after miR-136-5p inhibition, while there was no effect on other potential target genes (Figure 8A). Therefore, in the subsequent study we concentrated on revealing the relationship between miR-136-5p and the target gene Ino80. And luciferase assay confirmed that miR-136-5p could bind to Ino80 (Figure 8C). This suggests that Ino80 is indeed a direct target gene of miR-136-5p. In addition, we observed the changes Ino80 expression was upregulated in CMs after LED-Red illumination (Figure 8D, E). Next, we combined the application of LED-Red and miR-136-5p mimic to treat CMs. The results showed that the inhibitory effect of LED-Red on Ino80 RNA and protein expression was attenuated after miR-136-5p mimic pretreatment (Figure 8F, G). More importantly, Ino80 siRNA inhibited CMs proliferation, while overexpression of Ino80 in cardiomyocytes significantly promoted the proliferation rate of cardiomyocytes (Figure 8H, I and Figure S3A, B). Therefore, the above results suggest that Ino80 can bind to miR-136-5p as a downstream target of LED-Red to regulate CM proliferation.

## Discussion

In this study, we found that 630 nm LED as a low-power PBM therapy can promote post-infarction myocardial proliferation and revascularization, but without significant modulation of CFs. The physiological effect was mainly regulated by the activation of the downstream target gene Ino80 through the photo-sensitive miR-136-5p in CMs induced by LED-Red irradiation.

MI remains the leading cause of cardiovascular diseases all over the world 19. Because mammalian CMs are unable to renew themselves, the current medical strategies for MI are primarily focused on controlling and delaying the progression of heart failure. However, there is increasing evidence that CMs can re-enter the cell proliferation cycle and proliferate after receiving exogenous disruptions 2, 20, 21. Yet, the technical means of regulating myocardial proliferation still need to be constantly improved. In the present study, we showed that PBM can be used as a technique to regulate the proliferation of CMs.

The mechanisms by which PBM affects biological processes are complicated. Different wavelengths of light modulation may produce diametrically opposed effects. Blue light (peaked at 470 nm) is mainly used to kill cancer cells and inhibit the invasion and metastasis of tumors 22. Green light (peaked at 560 nm) is thought to have a positive effect on the migratory movement of stem cells 23. And red light (peaked at 630 nm) is mostly used for tissue wound healing, skin inflammation and arthritis treatment 24-27. Red light induces differential alterations in 76 genes in wounds treatment 28. It mainly exerts repairing effects by regulating inflammatory factors, cell growth factors, ROS, and energy production metabolism 29, 30. This is mainly because red light itself produces photons that are less likely to scatter and more likely to penetrate tissues. Consistent with previous reports, we also found that red light at 630 nm plays an important role in repairing myocardial injury compared with other wavelengths of light. Appropriately timed LED-Red light exposure has a pro-proliferative effect on myocardium due to its light accumulation effect. This finding indicates that LED-Red exhibits the promising application in heart regeneration after MI.

MiRNAs have continuously been found to be involved in the regulation of CMs proliferation and myocardial injury repair 17. However, there are fewer studies on the dynamic changes of miRNAs in PBM, especially in myocardial tissues. We systematically detected the differential changes of miRNAs in CMs after LED-Red irradiation by miRNA sequencing analysis. We found the presence of photosensitive miRNAs in CMs, which indicated that LED-Red involvement in infarct tissue damage repair might be regulated by miRNAs. And overexpression of miR-21a-3p and miR-136-5p had the ability to inhibit CMs proliferation. More importantly, the effect of miR-136-5p on proliferation was more prominent. Likewise, previous studies also reported that miR-136-5p is able to suppress the proliferation of several kinds of cells such as renal cell carcinoma 31. However, this is the first time to reveal miR-136-5p as a photo-sensitive and pro-proliferative miRNA in CMs.

Ino80 nucleosome remodeling protein has unique structural features and is involved in a variety of functions including transcriptional regulation, DNA replication and repair 32. Ino80 has been reported to be involved in proliferation regulation in a variety of cancers 33, 34. Recently, it was found that the deletion of Ino80 in the embryonic heart hinders the process of myocardial proliferation, leading to myocardial dysplasia 35. Consistent with this, we found that the Ino80 can bind to the photosensitive miR-136-5p and participate in LED-Red regulation of CMs proliferation.

CMs proliferation can be regulated by a variety of biological factors, and the various regulatory factors do not exist independently of each other in a state of non-interference. Therefore, LED-Red affecting miRNA-136-5p-Ino80 is not the only target of photopromoted cardiomyocyte proliferation. The signaling cascade response between various RNAs and proteins in PBM for CMs proliferation still deserves further investigation in the future.

### Study limitations

In the present study, we did not validate the role of miR-136-5p/Ino80 in regulating myocardial proliferation at the animal level. More importantly, differences in physiology between lower animals and humans need to be considered in future studies, which may lead to differences in PBM parameters (time, power, etc.). This may require a change in light irradiation to invasive treatments in humans. Also, the issue of clinical light safety needs to be considered. In the next studies, it will be important to investigate whether PBM still has the same therapeutic effect in big animals (pigs, monkeys).

## Conclusion

Collectively, this study found that 630 nm LED-Red irradiation could promote CM proliferation and revascularization, reduce infarct area and improve myocardial contractile function in mice with MI. Led-Red may activate the proliferation cycle of CMs by inhibiting photo-sensitive miR-136-5p and promoting the expression of Ino80.

## Materials and Methods

### Animals

KunMing male mice weighting 22 - 25 g were purchased from the Experimental Animal Center of the Second Affiliated Hospital of Harbin Medical University. The mice were housed under conditions of constant temperature and controlled illumination in a 12 h light-dark cycle. Food and water were available ad libitum throughout the study. Animal experimental protocols were approved by the Institutional Animal Care and Use Committee of Harbin Medical University.

### Establishment myocardial infarction model

First, male mice were anesthetized with 3% isoflurane. The left descending coronary artery (LAD) was ligated with a 7/0 prolene suture. While the sham operated mice underwent the same handle without ligation of LAD. After one week of MI, echocardiography was employed to assess the MI model was successfully established.

### Echocardiography analysis

Left ventricular short-axis papillary muscle level M-mode curves were recorded for measuring LVEF (left ventricular ejection fraction), LVFS (left ventricular fractional shortening), LVIDd (left ventricular end diastolic diameter), LVIDs (left ventricular end systolic dimension), LVEDV (left ventricular end diastolic volume) and LVESV (left ventricular end systolic volume).

### Culture of neonatal mice cardiomyocytes

The experimental method was carried out mainly with reference to this protocol^36^. Briefly, neonatal mice hearts were cut into 1-2 cm^2^ sizes and digested with trypsin (Solarbio, China). The cell suspension was collected by centrifugation and incubated at 37 °C, 5% CO_2_, 95% air for 90 min. Finally, the cell ratio was adjusted by adding in Dulbecco's modified Eagle's medium (DMEM; Life Technologies, USA) supplemented with with 10% fetal bovine serum (FBS; Gibco, USA) and 1×penicillin and streptomycin (Life Technologies, USA) and the plates were spread for culture.

### Phototherapy protocol

#### Animal experiment

A 630 nm LED-Red with a spot area of 1 cm^2^ and a power density of 2.5 mW/cm^2^ and 5 mW/cm^2^ was used for 10 min. The hair on the left side of the chest of the mice was removed. The mice were fixed on the light panel, and the light source was placed between the 2 nd and 5 th ribs on the left side, and the light was applied closely to the skin. Light experiments were performed according to the grouping conditions, Sham and MI groups were treated with the same conditions, but without red light irradiation.

#### Cellular experiments

Similarly, the light wavelength, power density, and irradiation time are preset. The well plate was placed directly below the light source and receives light stimulation. Main light parameters: wavelength 630nm, spot area 10 cm^2^, power density 2.5 mW/cm^2^, irradiation time 10 min.

### Transfection

Ino80 small interfering RNA (siRNA), and pcDNA3.1(+)-Ino80 plasmid, which were purchased from GENERAL BIOSYSTEMS (China). MiR-136-5p mimics, negative control mimics (NC-mimic), miR-136-5p inhibitor and negative control inhibitor (NC-inhibitor) were purchased from GenePharma (China). Transfections were performed by using Lipofectamine RNAiMAX, X-treme GENE, Lipofectamine 2000 (Invitrogen, USA). Transfections have been performed according to the manufacturer's protocols.

### Real-time PCR (qRT-PCR)

Total RNAs were extracted from CMs by using TRIzol reagent (Invitrogen, USA). The RNA samples were then reverse transcribed to cDNA. SYBR Green PCR Master Mix (Applied Biosystems, USA) was used to perform the quantitative real-time PCR. The primers used in this study are listed in Table S1.

### Immunohistochemistry

Hearts were harvested and fixed in 4% paraformaldehyde. Sections were stained with Masson Trichrome (Solarbio, China) to evaluate the myocardial fibrosis area. The density of capillaries was determined by von Willebrand Factor (vWF) (Cell Signaling Technology, USA) staining.

### Immunofluorescence

The hearts were optimal cutting temperature compound and cut into 4 μm sections. CMs and tissues sections while frozen were fixed with 4% paraformaldehyde (Solarbio, China). Then, the cells were permeabilized with 0.4% Triton X-100 (Solarbio, China) in PBS, blocked with goat serum (Boster, China) and incubated with primary antibodies against phospho-Histone H3 (pH3; Millipore, USA), Ki67 (Abcam,UK), α-actinin (Abcam, UK), vimentin (Abcam, UK) at 4 °C overnight followed by Alexa Fluor 488- or Alexa Fluor 594-conjugated secondary antibody for 1h. The cells were counterstained with DAPI (Solarbio, China) to label the nuclei.

### Ethynyl-2deoxyuridine incorporation (EdU) assay

The CMs were incubated with 5-ethynyl-2'-deoxyuridine by using Apollo 567 *in vitro* Kit (Ribobio, China). Briefly, cells were incubated with 5-ethynyl-2'-deoxyuridine for 12 h and fixed with 4% paraformaldehyde at 37 °C for 15 min. Next, the Apollo mixture reagent was added to evaluate DNA synthesis, then counterstained with DAPI.

### Cell Viability Evaluation

In the CCK-8 assay, CMs were seeded in a 96-well plate coated with gelatin at a concentration of 1×10^4^ cells/well. After adhesion, the cells were exposed to the LED-Red under different conditions. The cells were kept in the dark with 10 μL CCK-8/100 μL fresh medium in each well for 30 min at 37 °C, and a microtiter plate reader (Bio-Rad, USA) was used to obtain the absorbance at 490 nm to evaluate relative cell viabilities.

### LIVE/DEAD cell staining

Viability/Cytotoxicity Kit (Invitrogen, USA) was applied according to the manufacturer's instruction. The working concentrations of calcein-AM and ethidium homodimer-1 were 2 μM and 0.5 μM, respectively. Images were acquired using a fluorescence microscope (Olympus, Japan).

### HUVECs Tube assay

The 10mg/ml cold Matrigel® (Corning, USA) was evenly spread on a 24-well plate and incubated in the incubator for 30min. 1.2*10^5 HUVECs was added to each well, and the LED-Red group was treated with red light, while the Non-LED group was not irradiated, other experimental conditions were the same as LED-Red. The tubes were incubated in the incubator, and the tube formation was observed under the microscope at 30min, 1h, 2h, 3h and 6h after irradiation and pictures were collected.

### Western Blot analysis

The total protein was extracted from CMs with RIPA lysis buffer (Beyotime, China), and a BCA assay (Beyotime, China) was performed to quantify the protein concentrations. The equal amount of protein samples was loaded and resolved on SDS-PAGE gel, and transferred to nitrocellulose membrane (Millipore, USA). The membranes were blocked in 5% milk and then reacted overnight with primary antibodies against Ino80, Dis3, Srsf1 and GAPDH (Proteintech, USA) at 4 °C. The membranes were finally incubated with secondary antibodies for 1 h at RT and scanned with Odyssey (LI-COR Biosciences, USA).

### Dual luciferase reporter assay

The Ino80-mutant constructs were then achieved by mutating the seed regions where they were binding to miR-136-5p sequences. Ino80 wild type (WT) and the mutant derivative devoid of the miR-136-5p binding site (Ino80-mut) were cloned with the downstream of the coding region of the luciferase gene in the psiCHECK-2 luciferase vector. Briefly, CMs were seeded in 24-well plate and either miR-136-5p mimic or its negative control was co-transfected respectively with Ino80-WT and Ino80-mut dual-luciferase 3'-UTR vector. The luciferase activity was measured using a Dual-Luciferase Reporter Assay System (Promega, USA). The relative luciferase activity was normalized to renilla luciferase activity.

### Statistics analysis

Data are expressed as the mean ± SEM. Student's t test was used for comparisons between two groups and one-way analysis of variance followed by Bonferroni corrected post hoc t test was used for multi-group comparisons (version 8.0, GraphPad Software Inc., USA). *P* value < 0.05 was considered statistically significant.

## Supplementary Material

Supplementary figures and table.
