# Supplementary material for: Photobiomodulation Drives MiR-136-5p Expression to Promote Injury Repair after Myocardial Infarction
Source: Int J Biol Sci. 2022 Apr 18;18(7):2980–93. doi: 10.7150/ijbs.71440 (PMC9066112; doi:10.7150/ijbs.71440)
Supplement: Supplementary file 1 — Supplementary figures and table. [file ijbsv18p2980s1.pdf]

Supplementary Figure 1

The TargetScan Mouse algorithm predicts target genes that bind to miR-136-5p and are associated with proliferation regulation.

Supplementary Figure 2

LED-Red illumination device diagram

Supplementary Figure 3

The effect of overexpression of Ino80 on CM proliferation was detected using (A) EdU, (B) pH3. Arrows indicate proliferative signals, n = 3, Scale bar = 20 μm. ***$P <$ 0.001vs. CTL. The data represent the mean ± SEM.

Supplementary Figure 1.

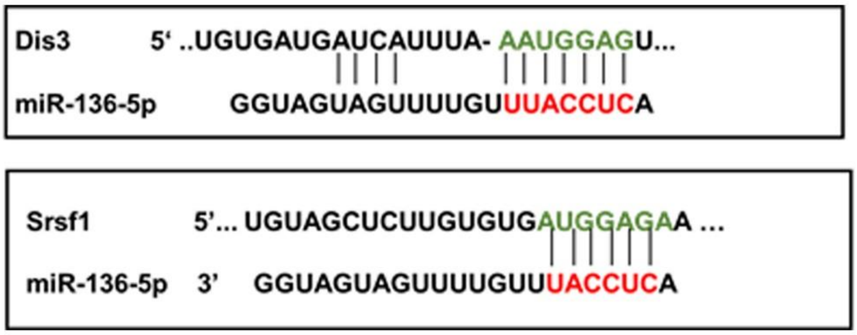

Supplementary Figure 2.

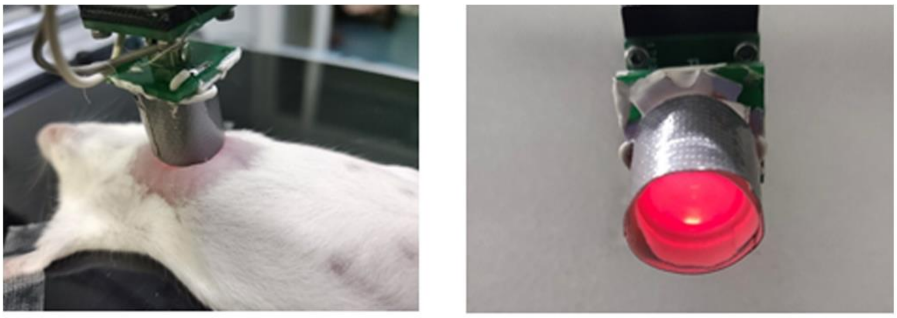

LED therapy

Supplementary Figure 3

A

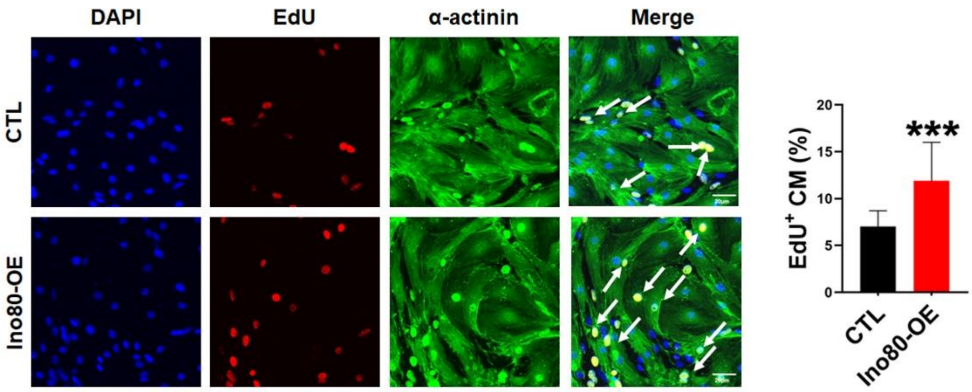

B

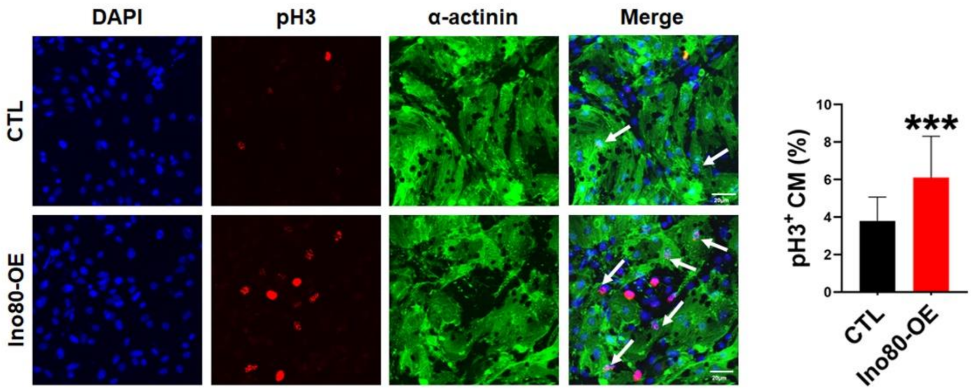

Table S1 Primer sequences

GENE	Forward primer	Reverse primer	Random primer
miR-451a	AAACCGTTACCATTA CTGAGTT	ATCCAGTGCAGGGT CCGAGG	GTCGTATCCAGTGCAGGGTCCGAGGT ATTCGCACTGGATACGACAACTCA
miR-466i-5p	TGTGTGTGTGTGTGTG TGTG	ATCCAGTGCAGGGT CCGAGG	GTCGTATCCAGTGCAGGGTCCGAGGT ATTCGCACTGGATACGACCACACA
miR-690	AAAGGCTAGGCTCAC AAAAA	ATCCAGTGCAGGGT CCGAGG	GTCGTATCCAGTGCAGGGTCCGAGGT ATTCGCACTGGATACGACTTTGGT
miR-6933-5p	TGGCGGCAGTCAGGA TACTT	ATCCAGTGCAGGGT CCGAGG	GTCGTATCCAGTGCAGGGTCCGAGGT ATTCGCACTGGATACGACACAGGT
miR-7226-5p	GCCAGGGAAGTTGAT TGGGAAG	ATCCAGTGCAGGGT CCGAGG	GTCGTATCCAGTGCAGGGTCCGAGGT ATTCGCACTGGATACGACCCCTTC
miR-135b-5p	TATGGCTTTTCATTCC TATGTG	ATCCAGTGCAGGGT CCGAGG	GTCGTATCCAGTGCAGGGTCCGAGGT ATTCGCACTGGATACGACGCCATA
miR-136-5p	CACTCCATTTGTTTTG ATGATG	ATCCAGTGCAGGGT CCGAGG	GTCGTATCCAGTGCAGGGTCCGAGGT ATTCGCACTGGATACGACTGGAGT
miR-219a-5p	CCTGATTGTCCAAAC GAATTT	ATCCAGTGCAGGGT CCGAGG	GTCGTATCCAGTGCAGGGTCCGAGGT ATTCGCACTGGATACGACCAATCA
miR-21a-3p	CAACAGCAGTCGATG GGTT	ATCCAGTGCAGGGT CCGAGG	GTCGTATCCAGTGCAGGGTCCGAGGT ATTCGCACTGGATACGACCTGTTG
miR-30a-5p	TGTAAACATCCTCGA CTGGAAG	ATCCAGTGCAGGGT CCGAGG	GTCGTATCCAGTGCAGGGTCCGAGGT ATTCGCACTGGATACGACTTTACA
Ino80	GCAAAGCCCCTTTAT CTTCAGT	CCAGAGGTTTCGCC AAGCAA	
